# Supplementary figures and images for: Ultrastructural alterations in the retinal pigment epithelium and photoreceptors of a Stargardt patient and three Stargardt mouse models: indication for the central role of RPE melanin in oxidative stress
Source: PeerJ. 2018 Jul 19;6:e5215. doi: 10.7717/peerj.5215 (PMC6054867; doi:10.7717/peerj.5215)

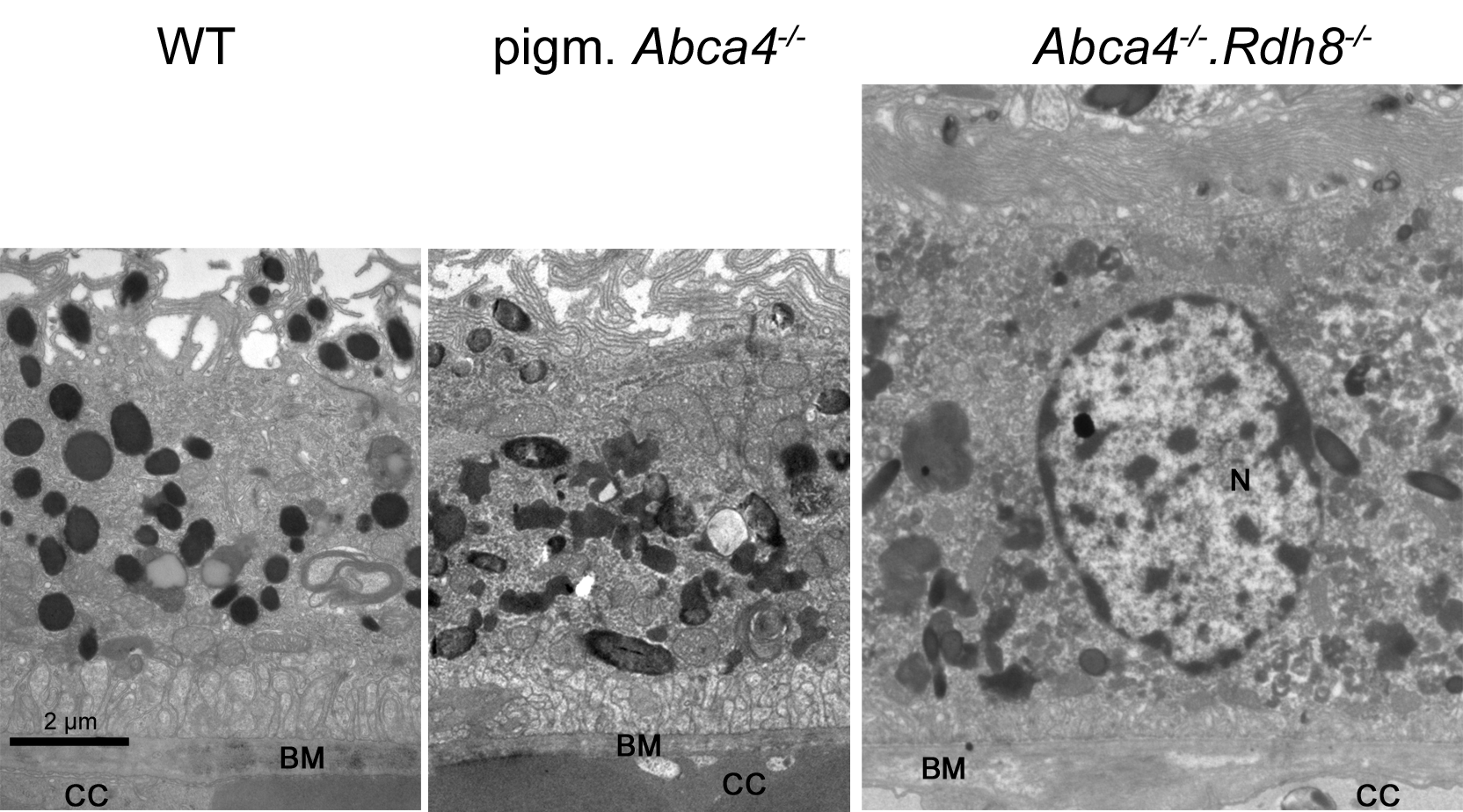

Supplement: Figure S1 — There is no apparent difference in cell size between pigmented WT and Abca4−∕−, but RPE in Abca4−∕−.Rdh8−∕− mice is hypertrophic. [file peerj-06-5215-s001.png]

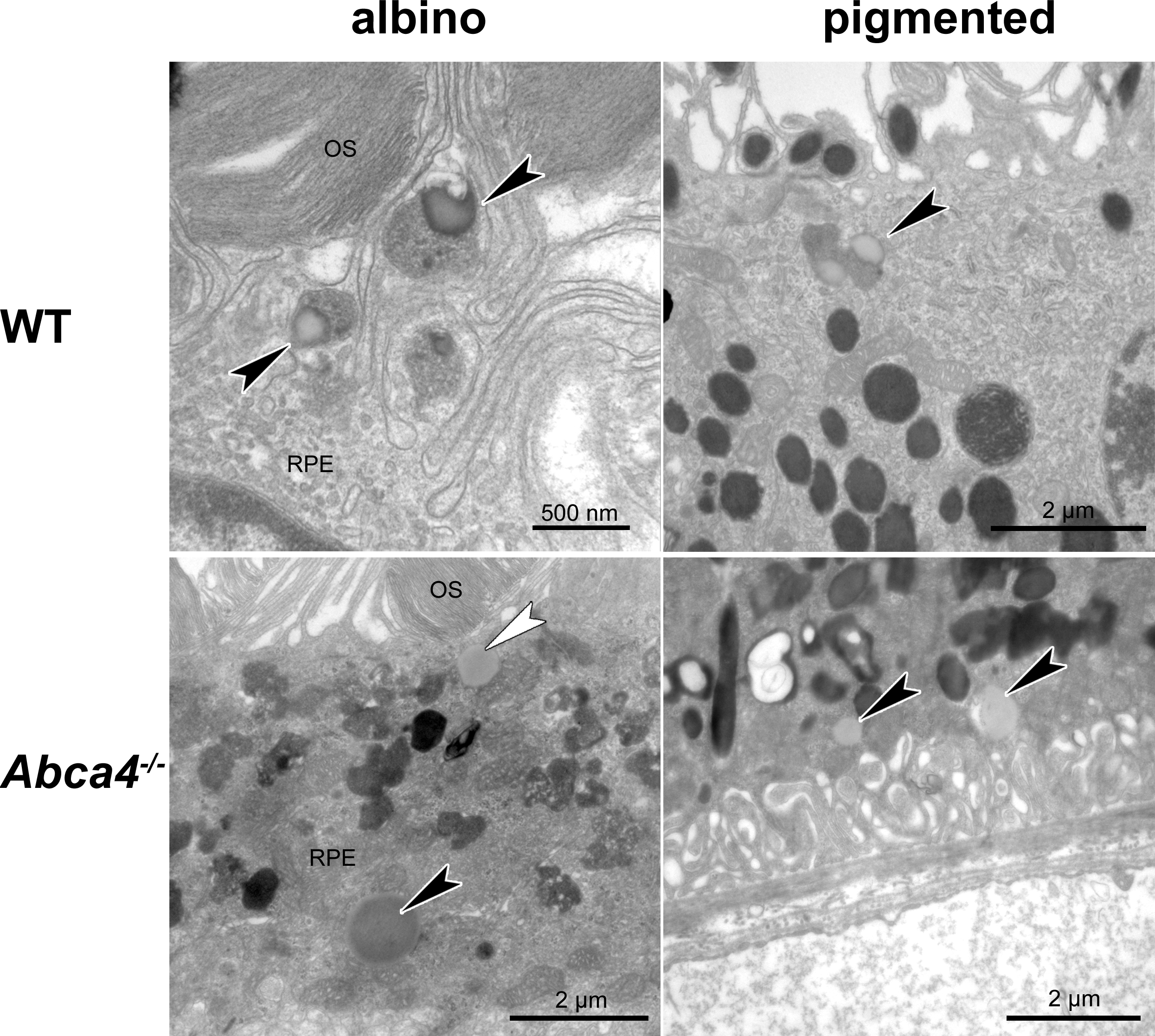

Supplement: Figure S2 — (Top) In WT animals, lipid droplets (black arrowheads) often fused with lipofuscin and were regularly located in the apical part of the RPE (left: albino WT, 4 months; right: pigm. WT, 12 months). (Bottom) Lipid droplets were regularly seen close to the basal labyrinth (black arrowheads) in albino Abca4−∕− mice and occasionally in pigmented Abca4−∕− mice. Rarely, lipid droplets were also seen apically close to the microvilli in albino Abca4−∕− mice (white arrowhead) (left: albino Abca4−∕−, 15 months; right: pigm. Abca4−∕−, 18 months). [file peerj-06-5215-s002.png]

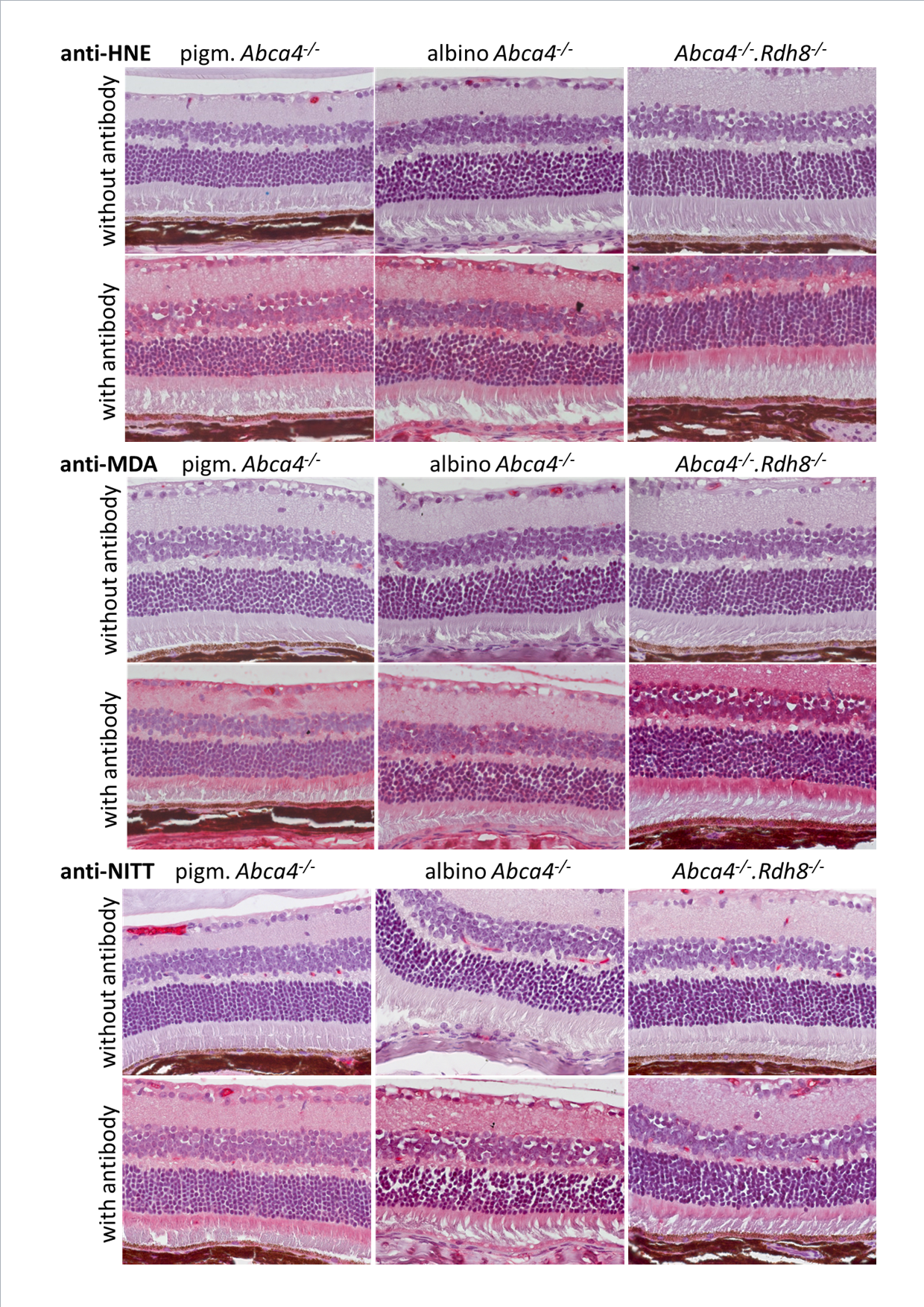

Supplement: Figure S3 — Upper panels for each primary antibody show negative controls (primary antibody was omitted); bottom panels for each primary antibody show the respective antibody stain (red). [file peerj-06-5215-s003.png]

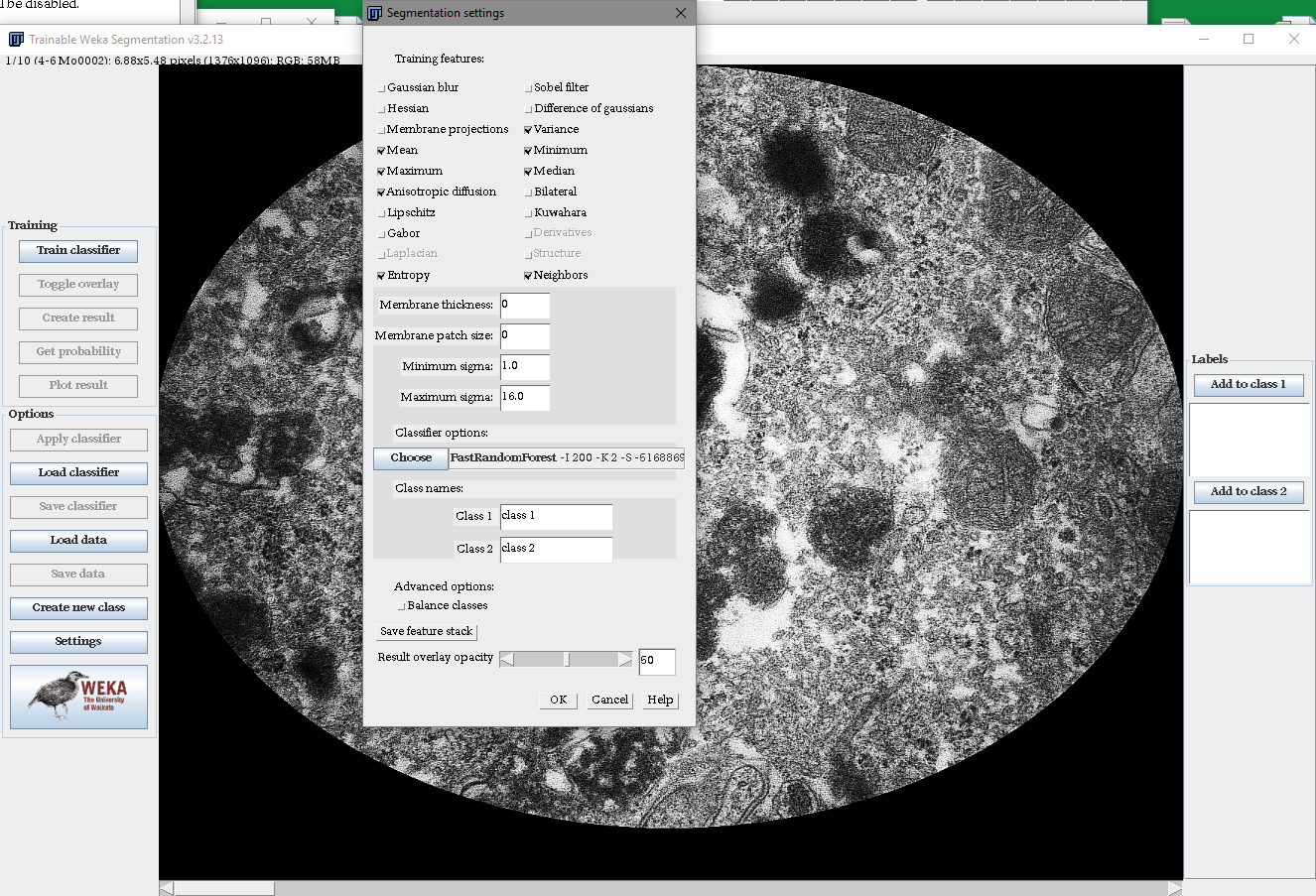

Supplement: Supplemental Information 6 — A screenshot showing the settings used to implement the segmentation. In the background, an exemplary electron micrograph is visible. Note that only an oval region of interest in the center of each micrograph was used for analysis to compensate for any vignetting that occured during image acquisition. [file peerj-06-5215-s009.png]
